# Polystyrene adsorbents: rapid and efficient surrogate for dialysis in membrane protein purification

**DOI:** 10.1038/s41598-020-73522-1

**Published:** 2020-10-01

**Authors:** Santosh Kumar Palanirajan, Punitha Govindasamy, Sathyanarayana N. Gummadi

**Affiliations:** Applied and Industrial Microbiology Laboratory, Department of Biotechnology, Bhupat and Jyoti Mehta School of Biosciences, Indian Institute of Technology Madras, Chennai, 600 036 India

**Keywords:** Biochemistry, Analytical biochemistry

## Abstract

Membrane protein purification is a laborious, expensive, and protracted process involving detergents for its extraction. Purifying functionally active form of membrane protein in sufficient quantity is a major bottleneck in establishing its structure and understanding the functional mechanism. Although overexpression of the membrane proteins has been achieved by recombinant DNA technology, a majority of the protein remains insoluble as inclusion bodies, which is extracted by detergents. Detergent removal is essential for retaining protein structure, function, and subsequent purification techniques. In this study, we have proposed a new approach for detergent removal from the solubilized extract of a recombinant membrane protein: human phospholipid scramblase 3 (hPLSCR3). N-lauryl sarcosine (NLS) has been established as an effective detergent to extract the functionally active recombinant 6X-his- hPLSCR3 from the inclusion bodies. NLS removal before affinity-based purification is essential as the detergent interferes with the matrix binding. Detergent removal by adsorption onto hydrophobic polystyrene beads has been methodically studied and established that the current approach was 10 times faster than the conventional dialysis method. The study established the potency of polystyrene-based beads as a convenient, efficient, and alternate tool to dialysis in detergent removal without significantly altering the structure and function of the membrane protein.

## Introduction

Membrane proteins (MPs) represent ~ 30% of the coding region in an organism’s genome. MPs predominantly occupy the membrane of a cell or an organelle and are involved in several vital cellular functions such as nutrients uptake, cell–cell interaction, signal transduction, and so on. The wide range of MPs’ function makes them as potential drug targets but less is known about its structure which is evident from the fact that only ~ 2% of the deposited structures in protein data bank are of MPs^1,2^. Deciphering the molecular mechanism involved in the host of functions rely on structural studies which in turn require MPs in large quantities. The difficulties associated with MPs solubilization and purification are the major hurdles in performing structural studies. Purifying MPs is challenging due to low endogenous expression, higher aggregation potency, and stability issues. The choice of detergent is pivotal in any MP purification process as it might perturb the structure and associated functions of the protein and the research is evolving towards custom made modular detergents^3,4^. The existing studies revealed that the most widely used conventional detergents such as lauryldimethylamine-N-oxide (LDAO), n-octyl- β -D-glucopyranoside (OG) and N-Dodecyl-β-D-maltoside (DDM) are replaced with novel detergents such as amphiphilic polymers (amphipols), nanodiscs, cholesterol-based amphiphiles, lipopeptide detergents, tripod amphiphiles (TPAs) and maltose-neopentyl glycol amphiphiles (MNGs) for better solubilization and increased stability of the MPs^5–9^. An alternate approach where MPs are isolated using SMALPs instead of detergents which are styrene-based styrene-maleic acid copolymers (SMA) lipid particles^10–15^. Each of these alternatives has its advantages and limitations, either they have to be customized depending on the nature of MPs or might result in decreased stability or increased chances of aggregation or precipitation in the presence of di or polyvalent cations and or pH changes^14,16^. The choice of detergents depends on the protein under study and the consequent experiments involved with the MPs. In general, to effectively solubilize the MPs from the protein-containing membrane extracts several parameters are to be optimized which include concentrations of detergents, incubation periods, buffer concentrations, temperature conditions, and salt concentrations. A higher concentration of detergents is generally required to extract the MPs and the presence of excess detergent could potentially affect the stability or interfere with further purification techniques. Hence the excess detergent is removed or replaced with an alternate detergent before subsequent purification^1,17^. A complete exchange or removal of detergent is generally attained by using chromatographic supports, where extensive washing with appropriate buffers containing necessary detergent is vital or by dialysis, where the removal or exchange is done with appropriate buffers or by ultra-filtration using high molecular weight cut-off membranes. Detergent removal by chromatographic support or by ultra-filtration requires a higher volume of exchange buffer with a higher probability of aggregation of MPs and is extensively labor-intensive. Dialysis apart from higher volumes of buffer requirement is relatively a slow process whose efficiency depends on the critical micelle concentration (CMC) of the detergent^17^.

In this study, we have used human phospholipid scramblases (hPLSCRs) as a model protein. hPLSCRs are a family of single-pass transmembrane proteins involved in phospholipids (PLs) translocation, cell proliferation, tumor suppression, antiviral response, and apoptosis^18^. Higher yields of pure protein could be facilitated by over-expressing the protein as a recombinant protein using the *E. coli* expression system. Overexpression of hPLSCRs (6 × Histidine Tag) in *E. coli* resulted in localization of hPLSCRs to inclusion bodies (IBs). From our previous studies, we have identified that N-Lauryl sarcosine (NLS), a mild anionic detergent improved the recovery of hPLSCR from IBs to 50% at a concentration of 0.3% in 4 h^19^. The NLS treated fraction is subjected to immobilized metal affinity chromatography (IMAC) where His tag binding to Ni–NTA matrix is affected in the presence of NLS. Therefore, NLS is currently exchanged with Brij-25 by conventional dialysis using 6 L buffer in a span of 36 h and is a major hiccup in the purification procedure. Detergent removal by polystyrene beads based hydrophobic adsorption has been rarely used in crystallization studies owing to faster or incomplete detergent removal or adsorption of protein on to beads^20^. Mostly the polystyrene beads were effectively used for the removal of non-ionic detergents with low CMC values whereas the present work has been intended at investigating the optimal conditions for adsorption of NLS, anionic detergent with very high CMC value (14.57 mM) onto Bio-Beads SM2 (Bio-Rad Laboratories). The current study is focused on evaluating the efficacy of this strategy for the purification of membrane proteins as an alternate tool for conventional dialysis. Therefore to proceed further with the optimization, the recombinant 6 × His-hPLSCR3 cloned in pET28a( +) is used and the insoluble inclusion bodies are separated and solubilized with 0.3% NLS as reported earlier^19,21^. The soluble fraction was added with the Bio-Beads SM2 for detergent removal while the fraction subjected to conventional dialysis served as the control experiment (Supplementary Fig. S-1).

## Results

### The adsorption capacity of Bio-Beads SM2

**Figure 1:**
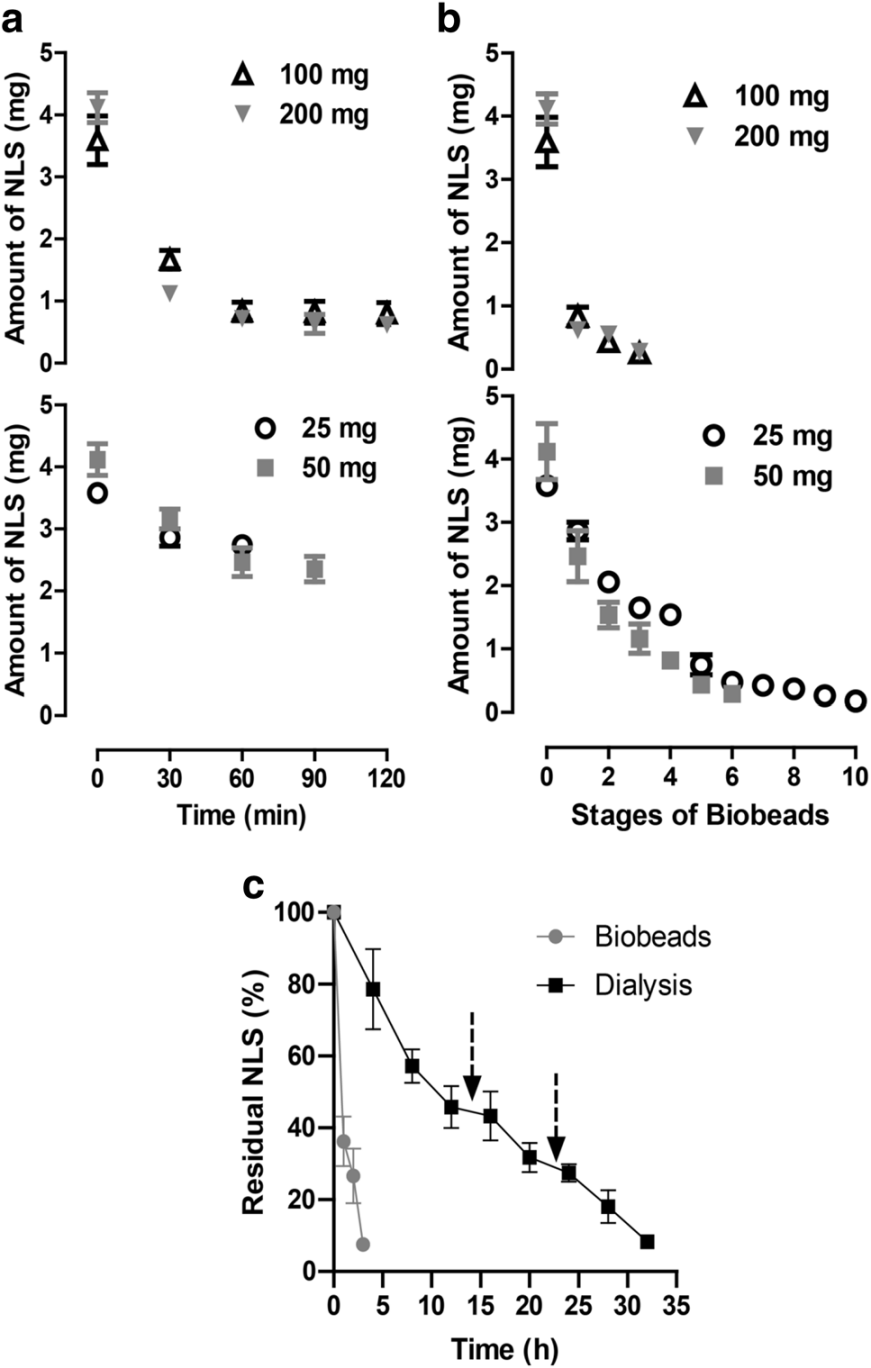
The adsorption capacity of Bio-Beads SM2 for N-Lauryl Sarcosine (NLS). Aliquots of 1 ml of 0.3% NLS solution were treated with 25 mg, 50 mg, 100 mg, and 200 mg of Bio-Beads SM2. The beads were continuously stirred and aliquots from the supernatant were removed at specific time intervals for the determination of the amount of NLS. (**a**) Time course of NLS removal by different amounts of initial beads. (**b**) The number of stages/ batches of beads addition required for the complete removal of NLS. (**c**) Comparison of NLS removal kinetics between dialysis and Bio-Beads SM2. Arrows indicate the time points of buffer change.

Figure 1 represents the adsorptive capacity of beads. Figure 1a depicts the time course of detergent removal by various weights of beads from the solubilization buffer containing hPLSCR3 along with 0.3% NLS. It is evident from the results that there is an optimal detergent-to-bead ratio beyond which the removal cannot be improved. The amount of NLS removed reached a plateau at about 60 min with different amounts of bio-beads and beyond which there was no further removal. The adsorbed detergents were leached out upon extending the incubation period beyond the plateau i.e., in case of 50 mg of beads addition, the amount of NLS slightly increased beyond 90 min of incubation which could be the result of very minimal desorption of the bound detergent from the beads to the solution. The adsorptive capacity of bio beads was estimated to be 0.02–0.03 mg of NLS/mg beads.

### Kinetics of detergent removal

The different amounts of beads were added in batches for complete removal of NLS from the protein solution and fresh beads were added at a regular interval of 60 min in respective amounts and the no of stages for complete removal was estimated (Fig. 1b). 10 batches of 25 mg beads were to be added in a span of 6 h for complete removal of NLS while 100 mg and 200 mg beads required only 3 batches. These results demonstrated the detergent adsorptive capacity of the Bio-Beads SM2 and also if the batch time was extended beyond 3 h, aggregation of protein molecules was higher, therefore to prevent aggregation, the NLS supernatant containing hPLSCR3 was diluted (1:1) with the buffer containing 0.025% Brij-25. The kinetics of NLS removal from the diluted protein solution between conventional dialysis and the beads based adsorption were analyzed and it was estimated that beads were able to completely remove NLS at a ~ tenfold higher rate than conventional dialysis (Fig. 1c).

### Purification of hPLSCR3

**Figure 2:**
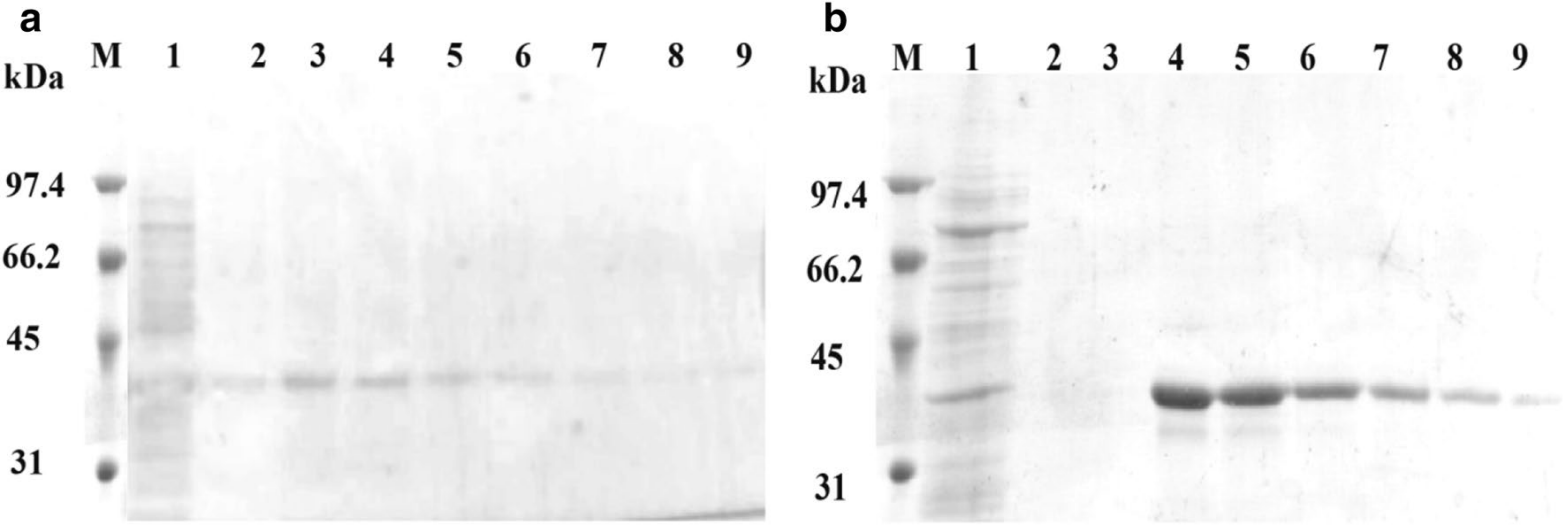
Purification of recombinant WT-hPLSCR3. (**a**) Ni–NTA purification of 6X His Tag WT-hPLSCR3 obtained after NLS removal by using Bio-Beads, Lane M-Marker, Lane 1—flow-through (after Ni–NTA binding), Lane 2—wash with 50 mM imidazole from Ni–NTA column, Lanes 3–9—purified WT-hPLSCR3 (elution with 250 mM Imidazole from Ni–NTA column). (**b**) Ni–NTA purification of 6X His Tag WT-hPLSCR3 obtained after NLS removal by dialysis, Lane M-Marker, Lane 1—flow-through (after Ni–NTA binding), Lane 2—wash with 50 mM imidazole from Ni–NTA column, Lanes 3–9—purified WT-hPLSCR3 (elution with 250 mM Imidazole from Ni–NTA column).

As evident from the Supplementary Figs. S-2 and S-3, the overexpressed hPLSCR3 was localized in the insoluble inclusion bodies (lane 3) which was recovered by NLS treatment (lane 5). After detergent removal from the NLS supernatant fraction by the Bio-Beads and dialysis approach (lane 6 of Supplementary Figs. S-2 and S-3 respectively), there was no significant loss of the target protein (Table 1). The recombinant hPLSCR3 was purified to homogeneity from NLS supernatant by Ni^2+^–NTA matrix-based immobilized metal affinity chromatography (lane 9 of Supplementary Figs. S-2 and S-3). It was evident from lane 1 of Fig. 2a,b that the target protein was tightly bound to the Ni^2+^–NTA resin and did not elute out in the flow-through fraction. The results implied that the anionic detergent, NLS was completely removed by the Bio-Beads approach similar to that of dialysis which otherwise would have impaired the binding of recombinant hPLSCR3 to the Ni^2+^–NTA matrix. Figure 2a,b revealed that the bound recombinant hPLSCR3 was obtained in the final elutes (lanes 3–9) of the Ni^2+^–NTA column as a homogenous protein. The amount of protein was estimated by the BCA assay method which revealed that there was no loss in the amount of protein before and after NLS removal by both the approaches as shown in Table 1. The estimated amount of purified hPLSCR3 (~ 7.8 mg) suggested that there was no significant difference in the yield of the target protein between dialysis and Bio-Beads based approach.

**Table 1:** Recovery and purification of human phospholipid scramblase 3 (hPLSCR3) from inclusion bodies (IBs) of *E. coli* Rosetta (DE3).

Stages of purification	Amount of protein (mg)	
	Dialysis	Bio-beads SM2
NLS supernatant (soluble fraction obtained after 0.3% NLS treatment for 4 h at 16 °C)	22.80 ± 3.5	
Soluble fraction after NLS removal	21.13 ± 2.5	20.37 ± 2.3
Purified hPLSCR3 (eluted after Ni^2+^–NTA chromatography)	7.70 ± 1.4	7.80 ± 0.3

### Intrinsic tryptophan Fluorescence

**Figure 3:**
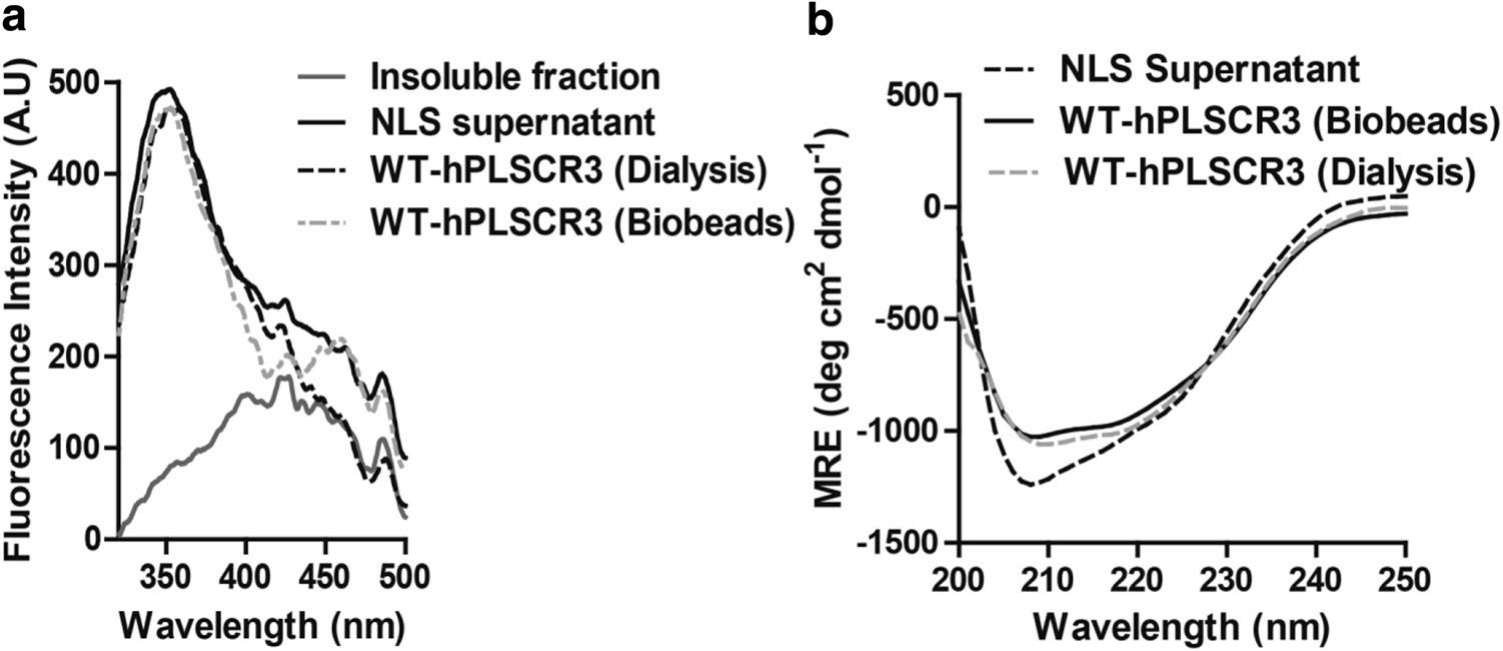
Secondary structural studies of recombinant WT-hPLSCR3. A comparison between hPLSCR3 obtained from dialysis and Bio-Beads SM2 based detergent removal. (**a**) Intrinsic tryptophan fluorescence studies of recombinant WT-hPLSCR3. Intrinsic fluorescence of the inclusion bodies, supernatant fraction obtained from IBs solubilized in 0.3% NLS, purified hPLSCR3 obtained after detergent removal by dialysis and by using bio-beads. (**b**) Far-UV CD spectra of recombinant WT-hPLSCR3. The supernatant fraction obtained from IBs solubilized in 0.3% NLS, purified hPLSCR3 obtained after detergent removal by dialysis, and by using bio-beads.

The structural integrity of the purified hPLSCR3 after NLS removal by Bio-Beads was analyzed and compared against the hPLSCR3 obtained after NLS removal by dialysis. The intrinsic tryptophan fluorescence spectra of the protein fractions were measured by exciting the protein samples at 295 nm and the emission spectra were observed between 300 and 500 nm. Figure 3a, revealed that the intrinsic tryptophan fluorescence of the recombinant hPLSCR3 after refolding from the insoluble fraction remained intact and was not perturbed during the NLS removal procedure by beads. The fluorescence maxima for intrinsic tryptophan residues usually fall around 340 nm for properly folded proteins. The hPLSCR3 fraction after NLS treatment and the purified hPLSCR3 obtained after NLS removal by dialysis and beads (Fig. 3a) was around 340 nm implying the hydrophobic tryptophan residues were properly buried and the protein was properly folded while that of the insoluble fraction showed a redshift which could be the outcome of exposed tryptophan residues of the disordered proteins dominantly present in the inclusion bodies.

### Secondary structural studies

The secondary structure of the protein fractions was analyzed by far UV CD spectroscopy where the spectra of the sample were recorded between 200 and 250 nm. It was observed from Fig. 3b that the spectra for NLS supernatant fraction showed two distinct minima at 208 nm and 222 nm. The general characteristic spectra for proteins containing larger proportions of α-helical structure retain two distinct negative minima around 208 nm and 222 nm. The spectra of purified hPLSCR3 obtained after NLS removal by using Bio-Beads and dialysis showed two distinct minima at 208 nm and 222 nm with slightly reduced intensities compared to that of the NLS supernatant fraction. The results implied that the purified protein in both the cases retained its characteristic α-helical secondary structure implying that the beads based detergent removal did not alter or perturb the secondary structure of the target protein.

### Functional assay

**Figure 4:**
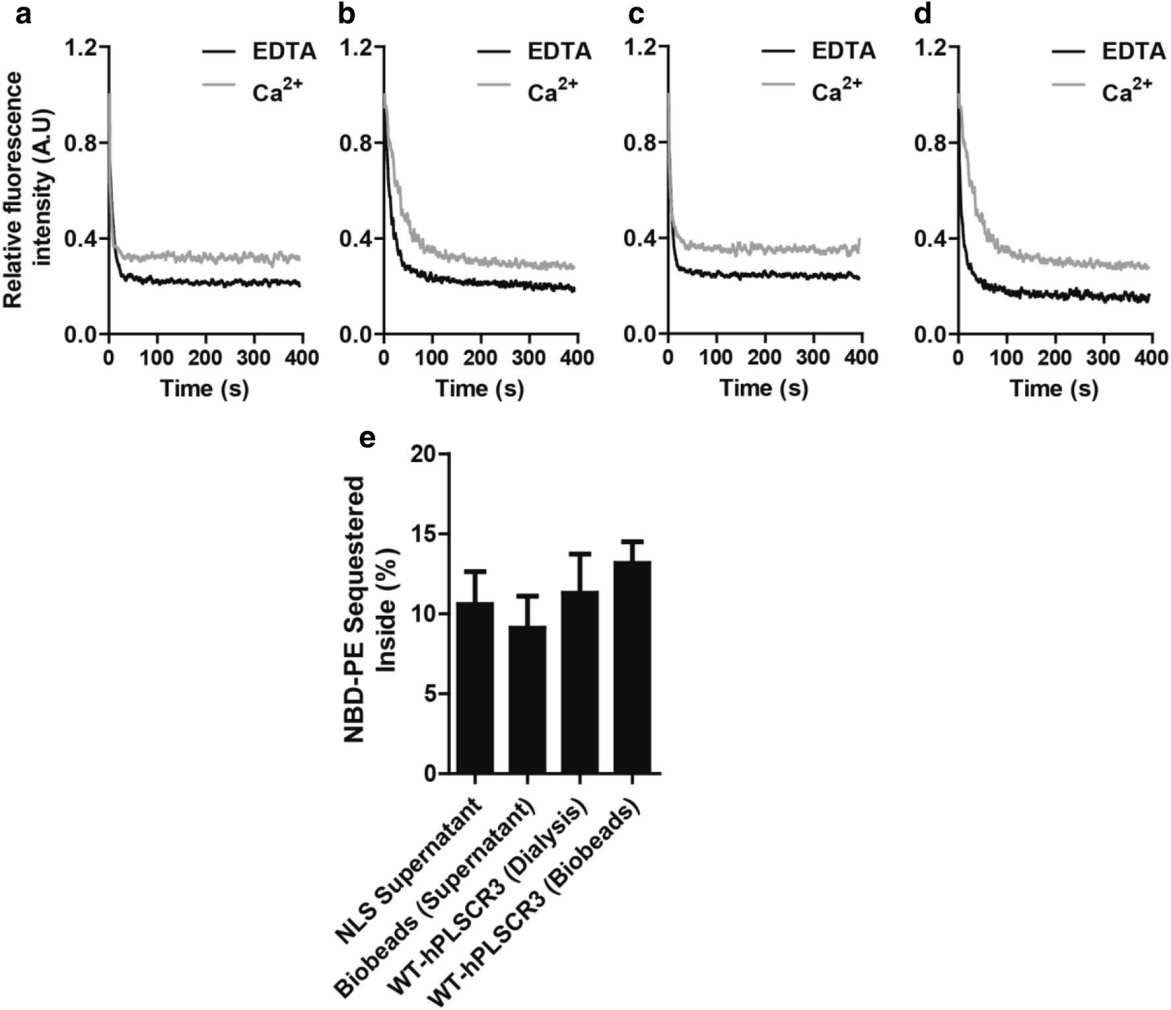
Functional assay. (**a**–**d**) Scramblase assay. The scramblase activity is measured by the fluorescence of NBD labeled PE and the plots represent the loss of fluorescence after dithionite (quencher) addition against time. Scramblase activity as a measure of NBD-PL sequestered. Representative plots for scramblase activity using outside labeled proteoliposomes reconstituted with (**a**) Supernatant fraction obtained from IBs solubilized in 0.3% NLS, (**b**) NLS supernatant after detergent removal by bio-beads, purified WT-hPLSCR3 obtained after detergent removal (**c**) by dialysis and (**d**) by using bio-beads. (**e**) Scramblase activity using proteoliposomes reconstituted with WT-hPLSCR3. Bar graphs representing the % of respective NBD-PE sequestered inside.

The functional activity of the protein fractions was estimated by the scramblase assay. A detailed scheme explaining the assay principle is provided in the Supplementary Fig. S-4. Briefly, sodium dithionite induced loss of fluorescence of NBD-conjugated lipids in the outer leaflet of artificial liposomes due to bleaching of the fluorophore is monitored with time. Scrambling of NBD-PL is represented by traces of proteoliposomes in the presence or absence of Ca^2+^. Our previous studies established that proteoliposomes reconstituted with active hPLSCR3 in the presence of Ca^2+^ facilitated translocation of the NBD-PE from outer leaflet to inner leaflet whereas, in the presence of EDTA, translocation was lacking. From the current structural studies, it was evident that the protein was structurally intact in the NLS supernatant as well as the purified fraction. Therefore, the hPLSCR3 containing protein fractions including the NLS supernatant, the detergent removed protein fraction (Bio-Beads), and the purified hPLSCR3 were reconstituted individually into different proteoliposomes of ~ 100–125 nm size (Supplementary Fig. S-5) labeled with NBD-PE. The hPLSCR3 was functionally active in all the fractions and was able to translocate PLs which is evident from Fig. 4a–d. The hPLSCR3 purified by this novel method was able to translocate ~ 10% of NBD labeled PE from the outer leaflet to the inner leaflet of the PL bilayer which is on par with the conventional purification (Fig. 4e). The functional assay with the direct reconstitution of hPLSCR3 containing NLS supernatant and the protein fraction after detergent removal by Bio-Beads showed that the recombinant hPLSCR3 was able to translocate ~ 9–10% of the NBD-PE similar to that of purified hPLSCR3 (Fig. 4e). The results advocate that the inclusion bodies also contain functionally active scramblase and if recovered efficiently and in sufficient amounts avoiding harsh chaotropic agents could be effectively used for further downstream processes and functional studies without the necessity of refolding.

## Discussion

Dialysis requires a minimum of 6 L buffer containing 0.025% Brij-25 for detergent exchange while beads based adsorption uses a minimal volume of buffer at a lesser time. Buffer change was done for every ~ 12 h when the NLS amount reaches equilibrium. We have shown from the course of our studies that the batch procedure for removing NLS from hPLSCR3 using Bio-Beads SM2 as a detergent removing agent provided a reproducible and faster way of complete NLS removal. The rapid removal of detergent is a mandate for highly unstable protein such as hPLSCR3 as the protein is highly unstable and prone for aggregation. A minimal decrease in the time required for purification might play a larger role in executing further downstream processes and studies with the protein. Further, NLS adsorption by beads in batch mode did not show loss of protein by adsorption or aggregation within the accuracy of protein estimation. Based on our results we conclude that the polystyrene-based Bio-Beads SM2 could be effectively used for complete and rapid removal of detergent in a membrane protein purification process. The approach will be an effective, cheaper, less laborious, and rapid method for detergent separation in membrane protein purification.

## Methods

### Overexpression of hPLSCR3

pET-28 a( +) plasmid containing WT-hPLSCR3 was transformed into *E. coli* Rosetta (DE3) strain. The transformed cells were grown in Luria Bertani broth (LB) containing kanamycin (50 mg l^−1^) and chloramphenicol (35 mg l^−1^) at 37 °C, 180 rpm. The cells were induced with 0.3 mM IPTG (IsoPropyl Thio Galactopyranoside) after the A_600 nm_ reaches 0.6–0.8 and grown for 6 h post-induction. T7 promoter was induced by the addition of IPTG and resulted in overexpression of (His6)-hPLSCR3 (6 × Histidine tag) in *E. coli* Rosetta (DE3) strain.

### Purification of hPLSCR3

The grown cells were then collected by centrifugation at 4 °C and 8500×*g* for 5 min. 1 g of The harvested cells were resuspended in buffer A containing 20 mM Tris–HCl, pH 7.4, 200 mM sodium chloride (NaCl) and to which 1 mM ethylenediaminetetraacetate (EDTA), 1 mM dithiothreitol (DTT) and 1 mM phenylmethylsulfonyl fluoride (PMSF) was added freshly before lysis (Lysis Buffer). For 1 g of harvested cells, 10 ml of lysis buffer was used. Cell lysis was carried out by probe sonication for 4 min with 4 s on/4 s off cycle at 30% amplitude. The lysate containing cells was then subjected to centrifugation at 4 °C and 13,000 xg for 30 min. The collected supernatant was labeled as soluble fraction while the pellet was labeled as an insoluble fraction, which had the inclusion bodies (IBs). The collected protein fractions were analyzed on 12% sodium dodecyl sulfate–polyacrylamide gel (SDS-PAGE) to trace the localization of overexpressed protein^21^.

### Solubilization of Inclusion Bodies (IBs)

The IBs in the insoluble fraction was separated as described above and was resuspended in 10 ml buffer A (20 mM Tris–HCl, 200 mM NaCl- pH 7.4). The insoluble fraction was added with 0.3% N-Lauryl Sarcosine (NLS). The resuspended solution was agitated at 16 °C, 110 rpm for 4 h^19^. Post incubation the resuspended solution was subjected to centrifugation at for 30 min at 13,000×*g* and 4 °C. The supernatant fraction was collected and labeled as NLS supernatant. The NLS was slowly removed and replaced with 0.025% (w/v) Brij-35 (buffer B) from NLS supernatant by step-wise dialysis using a 10-kDa cut-off membrane. NLS was completely removed within a duration of 36 h with a buffer change of 2 L buffer B for every 12 h^21,22^.

Briefly, the insoluble inclusion bodies containing the recombinant hPLSCR3 were solubilized in a buffer A containing 0.3% NLS. The soluble fraction obtained after detergent solubilization was subjected to dialysis for detergent exchange (control experiment)^21^. Alternately polystyrene beads commercially called as Bio-Beads SM2 (Bio-Rad Laboratories, USA) was added to remove the detergent for further purification^23^. NLS interferes with Ni–NTA binding and has to be removed before further process.

### Estimation of NLS

NLS was estimated by measuring the absorbance at 215 nm using UV-spectrophotometer (Shimadzu)^22,24^. A stock solution of NLS was prepared in buffer A and a standard graph was established with the observed absorbance values.

### Washing of Bio-Beads SM2

The polystyrene beads were thoroughly rinsed twice with methanol. After methanol, the beads were rinsed completely with deionized water and then with the buffer and the dry beads were discarded. In each washing step 1 g of beads were suspended in 25 ml of the respective solution and continuously stirred for 15 min at 25 °C. After washing wet beads were stored at 4 °C.

### Detergent adsorption by bio-beads

The beads after buffer wash were weighed and mixed to the protein solutions containing the detergent (NLS). The samples were subjected to gentle rotary spinning at 4 °C and aliquots were removed at regular time intervals to estimate the amount of detergent and protein present in the solution. The beads rapidly sediment in the absence of stirring as they are denser, thus facilitating the removal of supernatant from the mixture. Once the beads were saturated with the detergent i.e. beyond which the amount of detergent in the solution did not decrease, the supernatant was removed and added to another tube containing fresh beads. This is defined as one stage/batch. The adsorptive property of the beads allowed control over the detergent removal rate. The rate of detergent removal was lowered by adding small successive portions of beads instead of adding the entire amount of beads required for the complete removal of detergent all at once thereby preventing the aggregation of the membrane protein. The sudden removal of detergent resulted in protein solution turning viscous due to aggregation of protein and the protein could not be further purified. In the case of a protein solution of higher concentration or protein having higher aggregation potency or highly unstable protein solutions, it is better to dilute the solution before detergent removal by bio-beads.

### Purification of hPLSCR3

The Ni–NTA resin packed in a chromatography column was equilibrated with buffer B and the NLS removed protein solution was mixed with the resin for 4 h at 4 °C with continuous spinning at 10 rpm. After collecting the flow-through from the column, the Ni–NTA resin was sequentially washed with the Buffer B containing 20 mM and 50 mM imidazole to remove unbound proteins. The final elution of the bound target protein was done with Buffer B containing 250 mM imidazole. The eluted fraction containing target proteins were stripped of imidazole and metal-ions by dialyzing against buffer B, prepared from chelex-100 resin-treated water. The eluted fractions were analyzed using 12% SDS-PAGE.

### Protein estimation

The amount of protein was estimated by the bicinchoninic acid assay (BCA assay) method using bovine serum albumin (BSA) as standard^21^.

### Scramblase assay

#### Liposome preparation

Liposomes were prepared as previously described^25,26^. Briefly, Egg phosphatidylcholine (PC) and phosphatidylserine (PS) (Sigma Aldrich) were taken such that 4.5 μmol of total PLs were comprised of 90% Egg PC and 10% brain PS. The lipid mixture was dried under N_2_ and dissolved in reconstitution buffer containing 10 mM HEPES/NaOH (hydroxyethyl-1-piperazine ethanesulfonic acid/sodium hydroxide), pH 7.5, 100 mM NaCl, and 1% (w/v) Triton X-100. The detergent (Triton X-100) was slowly removed by Bio beads with 3 changes of 200 mg Bio-Beads SM2 (Bio-Rad) per ml of liposomes and incubated at 4 °C for 16 h with gentle rotation. The protein fraction was reconstituted into liposomes for functional assay and is termed proteoliposomes which were prepared similar to that of liposomes. To the dissolved lipid mixture, 100 µg of recombinant hPLSCR3 was added after the solubilization step and then detergent was slowly removed by Bio-Beads^19,21,22^.

#### Outside labeling

The beads were allowed to settle and then the supernatant fractions containing the liposomes and proteoliposomes were collected by centrifugation for 45 min at 230,000×*g* and 4 °C. (MLA130 rotor, Beckman Coulter ultracentrifuge, USA). The supernatant was discarded and the pellet fraction containing collected vesicles was dissolved in the assay buffer (10 mM HEPES/NaOH, pH 7.5, and 100 mM NaCl) and the centrifugation was repeated with the conditions mentioned above. The washing of the collected vesicles by centrifugation was repeated twice after the initial collection. The washed liposomes and proteoliposomes were extruded through a 100 nm polycarbonate membrane filter pre-equilibrated with the assay buffer to obtain uniform sized unilamellar vesicles. The extruded liposomes and proteoliposomes were then incubated at 37 °C for 5 min with 0.3 mol % of NBD − PE (7-nitrobenz-2-oxa-1,3-diazol-4-yl-phosphatidylethanolamine) (Avanti Polar Lipids, USA). The labeled liposomes and proteoliposomes were then collected and washed twice with assay buffer by centrifugation as described above. The labeled liposomes and proteoliposomes collected from the previous step were resuspended in 1 ml of assay buffer and were divided into two equal fractions. The divided fractions were incubated either in the presence of 3 mM EDTA or 3 mM Ca^2+^ at 37 °C for 4 h^19,21^.

#### Scramblase assay

Post-incubation the labeled liposomes or proteoliposomes were diluted and transferred to stirred fluorescence cuvettes for fluorescence measurement. Precisely ~ 50–100 µL aliquots of the liposomes or proteoliposomes were diluted to 2.5 mL with the assay buffer and the fluorescence intensity was continuously recorded for 600 s at 25 °C with constant stirring using a fluorescence spectrophotometer (Perkin-Elmer LS-55(Waltham, Massachusetts, US). Fluorescence intensity was measured by exciting the sample at 470 nm and emission intensity was recorded at 532 nm with slit widths of 10 nm (excitation) and 10 nm (emission). The fluorescence intensity was recorded for an initial 200 s, and after which the freshly prepared 20 mM dithionite (in 1 M Tris Base pH- 11.6) was added to the sample and the fluorescence was monitored for an additional 400 s. The fluorescence intensity decreased after the addition of the membrane-impermeable quencher (dithionite), and the residual fluorescence was recorded.

The residual fluorescence values after dithionite addition were normalized with the average of the initial fluorescence values observed for each sample. The difference in the fluorescence intensity between the non-quenchable fluorescence observed in the presence of Ca^2+^ and EDTA for each sample is attributed to the scramblase activity of the reconstituted protein fraction.

Scramblase activity was calculated as % NBD-PE translocated defined by.

$${\text{Scramblase}}\,{\text{activity}} = {\text{F}}_ {\text{Ca}^{2+}}- {\text{ F}}_{{{\text{EDTA}}}} ,$$

where F_Ca_^2+^ and F _EDTA_ are the non-quenchable relative fluorescence intensity in the presence of Ca^2+^ and EDTA respectively^19,21^.

#### Far-UV CD studies

Far-UV CD spectra of the buffer and protein solution were recorded using Jasco J-810 spectropolarimeter (Easton, MD, USA). The spectra were recorded with 10 μM protein concentration at a wavelength range of 200–250 nm. The measurements were done in a 0.1 cm path length cuvette at a scanning speed of 10 nm per min with a set bandwidth of 2 nm at 25 °C in a thermostat cell holder. The buffer spectra recorded under similar conditions were was subtracted from the sample spectra^21^.

#### Intrinsic tryptophan fluorescence

The intrinsic fluorescence spectra of the protein fractions were recorded in the fluorescence spectrophotometer (Perkin-Elmer LS-55). The fluorescence emission spectra were recorded by exciting the sample at 295 nm and the emission spectra were recorded between 300 and 500 nm. The measurements were carried out at 25 °C at a scanning speed of 100 nm per min, with the excitation and emission slit widths of 5 nm each.

## Supplementary information

Supplementary file 1.
