# Supplementary material for: Polystyrene adsorbents: rapid and efficient surrogate for dialysis in membrane protein purification
Source: Sci Rep. 2020 Oct 1;10:16334. doi: 10.1038/s41598-020-73522-1 (PMC7529760; doi:10.1038/s41598-020-73522-1)
Supplement: Supplementary file 1 [file 41598_2020_73522_MOESM1_ESM.docx]

**Supplementary Material to Bio-beads: Rapid and efficient surrogate for dialysis in membrane protein purification**

**Polystyrene adsorbents: Rapid and efficient surrogate for dialysis in membrane protein purification**

*Santosh Kumar Palanirajan, Punitha Govindasamy, and Sathyanarayana N Gummadi**

**Supplementary Protocol**

**Particle Size analysis**

Dynamic light scattering was used to analyze the size of the proteoliposomes reconstituted with different protein fractions. Dynamic light scattering was performed by using zetatrac particle size analyzer (USA) and the results were analyzed by Microtrac-FLEX software 10.5.3. Particle size analyzer measures the size distribution by the velocity of the particles dispersed in the buffer. The size of the proteoliposomes was monitored with a run time of 60 s and 15 repetitions.^1^

**References**

1. Palanirajan, S. K. & Gummadi, S. N. Heavy-Metals-Mediated Phospholipids Scrambling by Human Phospholipid Scramblase 3: A Probable Role in Mitochondrial Apoptosis. *Chem. Res. Toxicol.* **33**, 553–564 (2020).

**Supplementary Figures**

**
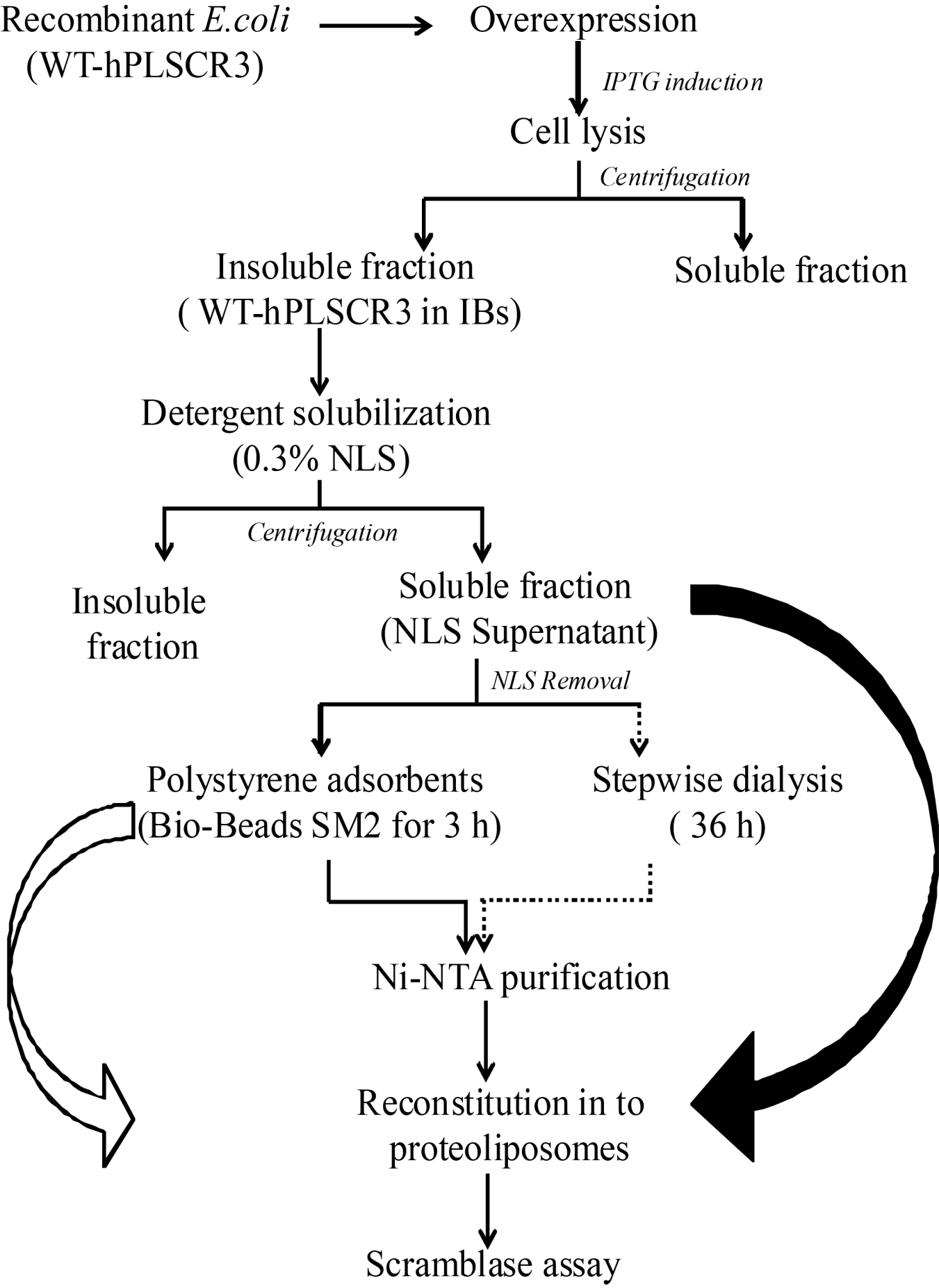
**

**Fig S-1. Schematic representation of the purification stages.**

The curved arrows represent the direct reconstitution of the protein fractions from the respective stages for functional (Scramblase) assay without purification.

**Fig S-2.** Ni-NTA purification of 6X His Tag WT-hPLSCR3 obtained after NLS removal by bio-beads. Lane M-Marker, Lane 1- induced cell lysate, Lane 2- soluble fraction (after cell lysis), Lane 3- Insoluble fraction; IBs (after cell lysis), Lane 4-insoluble fraction (after NLS treatment). Lane 5-soluble fraction/NLS supernatant (after NLS treatment), Lane 6- Diluted soluble fraction after NLS removal by bio-beads adsorption, Lane 7- Flow through, Lane 8- wash with 50 mM Imidazole from Ni-NTA column, Lane 9- Purified WTSCR3 (elution with 250mM Imidazole from Ni-NTA column).

**Fig S-3.** Ni-NTA purification of 6X His Tag WT-hPLSCR3 obtained after NLS removal by dialysis. Lane M-Marker, Lane 1- induced cell lysate, Lane 2- soluble fraction (after cell lysis), Lane 3- Insoluble fraction; IBs (after cell lysis), Lane 4-insoluble fraction (after NLS treatment). Lane 5-soluble fraction/NLS supernatant (after NLS treatment), Lane 6- Diluted soluble fraction after NLS removal by dialysis, Lane 7- Flow through, Lane 8- wash with 50 mM Imidazole from Ni-NTA column, Lane 9- Purified WTSCR3 (elution with 250mM Imidazole from Ni-NTA column).


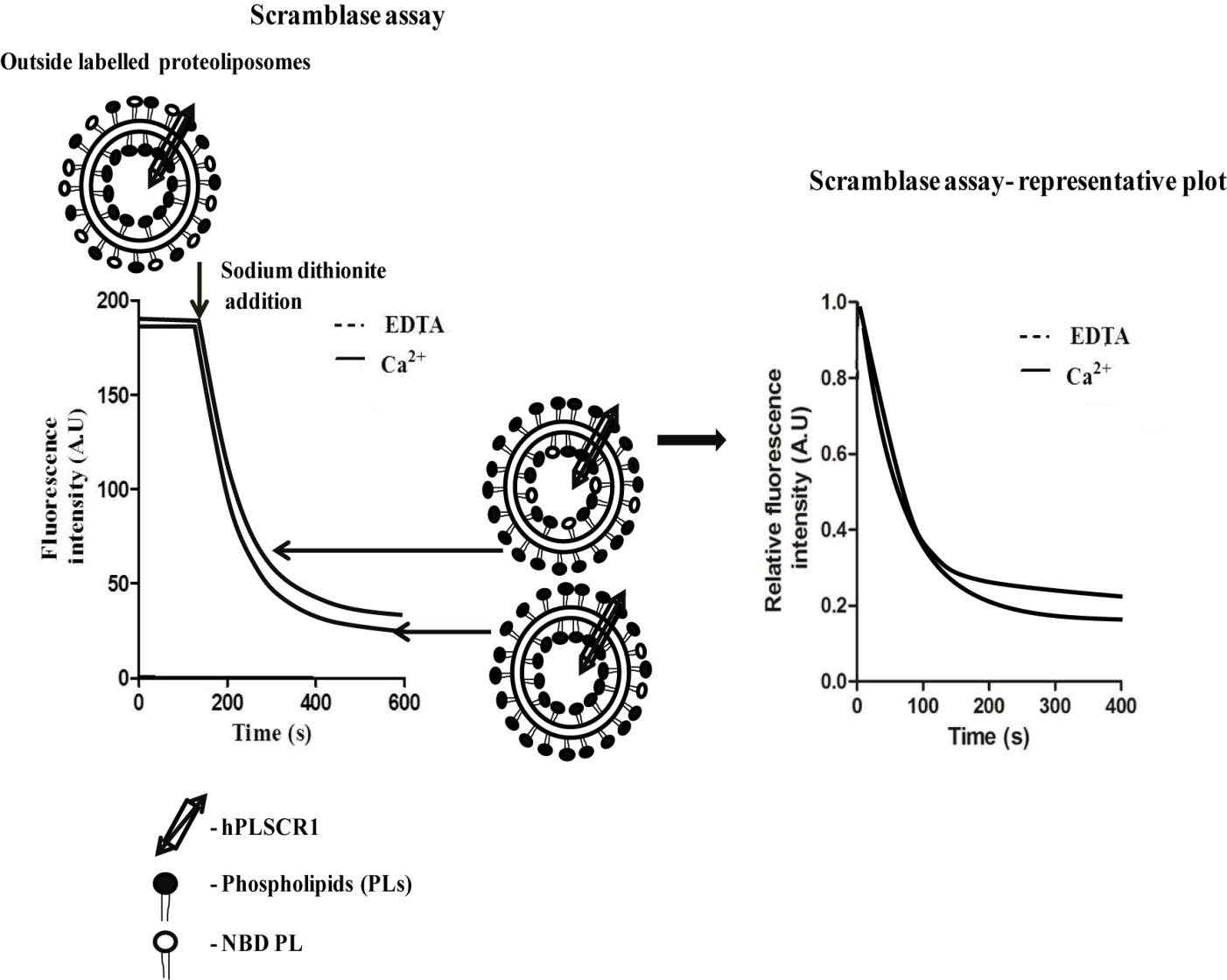


**Fig S-4. Schematic representation of the *in-vitro* scrambling assay.**

Scheme showing scramblase assay with outer leaflet labeling. Sodium dithionite induced loss of fluorescence of NBD-conjugated lipids in the outer leaflet of artificial liposomes due to bleaching of the fluorophore is monitored with time. Scrambling of NBD-PL is represented by traces of proteoliposomes in the presence or absence of Ca^2+^. Proteoliposomes reconstituted with active hPLSCR1 in presence of Ca^2+^ facilitates translocation of the NBD-PLs from outer leaflet to inner leaflet whereas, in presence of EDTA does not translocate the NBD-PLs. Upon dithionite addition, the NBD-PLs on the outer leaflet are quenched leading to a decrease in 80% of fluorescence. Since dithionite is membrane impermeable, the translocated NBD-PLs in the inner leaflet are protected, thereby retaining the fluorescence. The incorporated hPLSCR3 translocates bidirectionally and hence some of the NBD-PLs are back flipped leading to a further decrease in fluorescence. Hence in total ~70% of fluorescence is lost. The fluorescence intensity values after dithionite addition were normalized with initial fluorescence intensity (before dithionite addition) and the difference in residual fluorescence between Ca^2+^ treated proteoliposomes and EDTA treated proteoliposomes were represented as the activity of scramblase.

**
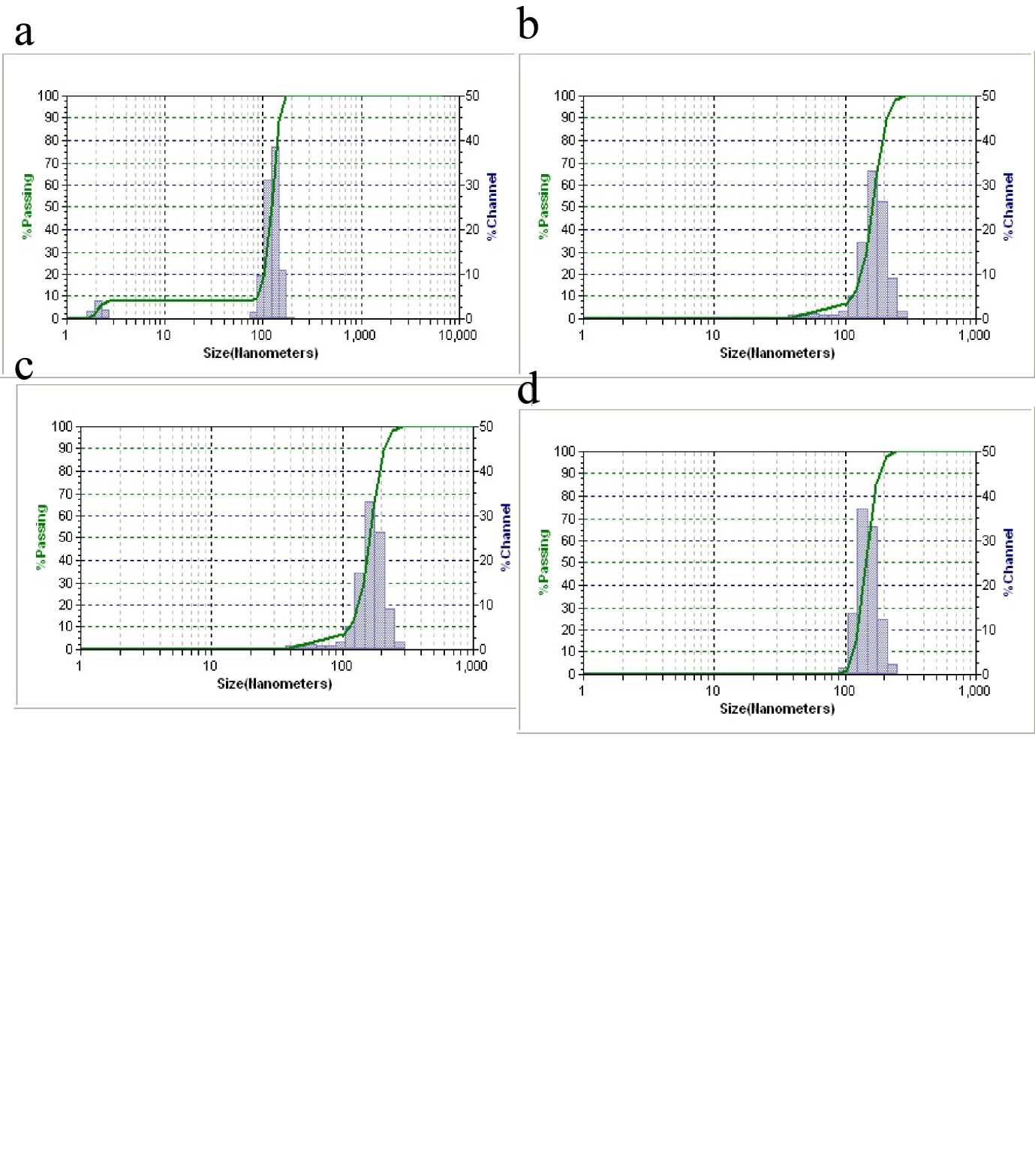
**

**Fig S-5. Reconstituted vesicles characterization**

Particle size analyzed by DLS for proteliposomes reconstituted with (a) NLS Supernatant, (b) WT-hPLSCR3 after NLS removal by biobeads, (c) WT-hPLSCR3 purified after NLS removal by dialysis, (d) WT-hPLSCR3 purified after NLS removal by biobeads. All the proteoliposomes had an average size of 100 nm implying the vesicles were not aggregated.
